# Early-onset neonatal infection in pregnancies with prelabor rupture of membranes in Kosovo: A major challenge

**DOI:** 10.4274/tjod.73600

**Published:** 2018-09-03

**Authors:** Vlora Ademi Ibishi, Rozalinda Isjanovska, Anne E. Malin

**Affiliations:** 1 University of Prishtina Faculty of Medicine, Department of Obstetrics and Gynecology, Prishtina, Kosovo; 2 Ss. Cyril and Methodius University Faculty of Medicine, Institute for Epidemiology, Biostatistics and Medical Informatics, Skopje, Republic of Macedonia; 3 Global Research Institute, Non-governmental Organization, Bishkek, Kyrgyzstan

**Keywords:** Prelabor rupture of membranes, early-onset neonatal infection, risk factors

## Abstract

**Objective:**

Prelabor rupture of membranes (PROM) is a common event in obstetrics that has a major impact in pregnancy outcome. This condition is linked to a number of pregnancy and birth complications with early-onset neonatal infection (EONI) being one of the major threats. This study was undertaken to determine the rate of neonatal infection in newborn infants with a maternal history of PROM and to evaluate the association of risk factors with neonatal infection following PROM.

**Materials and Methods:**

A cross-sectional descriptive design was used to analyze a population of 200 pregnant women presenting to the Obstetrics and Gynecology Tertiary Center in Kosovo (between 2013 and 2015) with PROM who gave birth to single newborns. Data including demographic characteristics, neonatal outcome, and risk factors for infectious neonatal morbidity were recorded and analyzed.

**Results:**

The study included 200 pregnant women with PROM and their newborns. Participant demographics included: the majority were young, aged between 20 and 29 years (67%), primiparous (67.5%), unemployed (92%), completed secondary level of education (83%), and with middle socioeconomic status (86%). Overall, 13% of the newborns had early-onset neonatal infection, and sepsis was proven in 5% of cases. Newborns of mothers with risk factors such as preterm (<37 weeks) PROM, low gestational weight at birth, prolonged rupture of membranes, maternal colonization, and low Appearance, Pulse, Grimace, Activity, Respiration score at birth had higher rates of infection compared with newborns of mothers without these risk factors.

**Conclusion:**

The rate of EONI in pregnancies complicated with PROM continues to be a global challenge in perinatology, and as this study reports, also a major challenge for Kosovo. Future research, revision and improvement on prenatal care and practices, timing of delivery, medical treatment, and prophylactic use of antibiotics in PROM are needed to reduce rates.

**PRECIS:** The purpose of this study was to determine the rate of neonatal infection in newborn infants with a maternal history of prelabor rupture of membranes and to evaluate association of risk factors with neonatal infection in pregnancies complicated with prelabor rupture of membranes.

## Introduction

Kosovo continues to have a high rate of early neonatal morbidity and mortality^(1)^. According to Azemi et al.^(2)^ and local annual perinatal reports, perinatal infections are one of the three main causes of early neonatal morbidity and mortality. There are, however, limited data about the rate and risk factors of early-onset neonatal infection (EONI) in pregnancies, including the focus of this study, EONI in pregnancies complicated with prelabor rupture of membranes (PROM), in Kosovo. PROM refers to rupture of the chorioamniotic membranes prior to the onset of labor and prior to the onset of clinically-apparent labor contractions. It can occur at any gestational age, thus this condition has been classified as “preterm PROM” or “term PROM”, depending on whether the event occurs before or after 37 weeks of gestation^(3)^. Early-onset infectious morbidities in newborn implies infections that occur from birth to seven completed days after birth, and these are considered to be one of the major threats in patients with PROM. The time interval between the rupture of membranes and onset of labor pain is called the latent period, and the time interval between the rupture of membranes and delivery is called the interval period (IP). The minimum latency for diagnosis of PROM is one hour. According to Caughey et al.,^(4)^ latency after membrane rupture is inversely correlated with gestational age at membrane rupture. At term, 50% of pregnancies complicated with PROM will go into labor spontaneously within 12 hours, 70% within 24 hours, 85% within 48 hours, and 95% within 72 hours. Among pregnancies complicated with preterm PROM, 50% will go into labor within 24 to 48 hours, and 70-90% within seven days.^(4)^ PROM is a common obstetric complication, but it is difficult to give an accurate incidence because of the wide variations reported in the existing literature. Recent data suggest that PROM occurs in approximately 10 to 12% of all pregnancies. Of these pregnancies, PROM complicates about 8% of term pregnancies and 2 to 3% of preterm pregnancies.^(4)^ A greater incidence of PROM was found in earlier research studies, which narrowed the PROM incidence range as 14 to 17%^(5)^. The etiology of PROM is almost certainly multi-factorial and in some cases unknown. The biologic mechanisms behind the development of PROM include intrinsic membrane weakness, mechanical stress, and ascending infection.^(6)^ PROM is linked to a number of adverse maternal and neonatal outcomes. The most frequent maternal consequences associated with PROM are chorioamnionitis, endomyometritis, wound infection, pelvic abscess, bacteremia, and postpartum hemorrhage.One of the most serious neonatal consequences associated with PROM is EONI. Physicians, and especially obstetricians, have long debated whether intrauterine infection is a cause or consequence of PROM and it seems likely both pathways are possible. The case for intrauterine infection being a consequence of PROM appears to be proved. Prior to rupture of membranes, the amniotic cavity is nearly always sterile. The physical properties of the intact placental membranes usually represent an effective barrier in preventing entry of bacteria. With rupture of membranes, protection of the fetus from the external micro-organisms ceases, bacteria from the lower genital tract typically enter the amniotic cavity, thus increasing the potential for subsequent maternal, fetal, and neonatal infection.^(7)^ To determine the rate of neonatal infection in newborn infants with a maternal history of PROM, and to evaluate the association between risk factors and neonatal infectious morbidity.

## Materials and Methods

This cross sectional descriptive study was conducted at the Tertiary Obstetrics and Gynecology Clinic University Clinical Center of Kosovo from September 2013 to July 2015. The study participants included pregnant women who were admitted with PROM. The selection of the study participants was based on the defined inclusive criteria: women with singleton pregnancy at or >28 weeks of gestation, presenting with PROM, and giving birth within 72 hours after PROM. Women with preexisting comorbidities and fetal anomalies were excluded from the study. A total of 200 pregnant women who presented with PROM who fulfilled the study’s inclusion/exclusion criteria and their newborns were included in the study informed consent was obtained from all potential participants. This study was approved by Research Ethics Committee of the Prishtina University, University Clinical Center of Kosovo (approval number 1451). A specific questionnaire and evaluation form was prepared and used to collect data prospectively at admission and thereafter. Data covering demographic characteristics and clinical data including gestational age at birth, gestational weight at birth, 1^st^ and 5^th^ minute Apgar (Appearance, Pulse, Grimace, Activity, Respiration) score, interval from PROM to delivery, maternal colonization, and early neonatal infectious morbidity were recorded and analyzed. Confirmation of gestational age was based on the last menstrual period, and in patients with irregular cycle or unknown last menstrual period, the gestational age was determined based on the medical records of the first trimester ultrasound examination. Confirmation of the diagnosis of rupture of membranes was documented through sterile speculum examination confirming the pooling of amniotic fluid in the posterior vaginal fornix or/and direct visualization of fluid leakage from the cervical canal. An ultrasound examination was performed to confirm fetal wellbeing. Immediately after delivery, the physical condition of the newborn was evaluated using Apgar scores by a neonatologist who was present at the birth. The newborns were observed during the first seven days of life during the early neonatal period. EONI was the main outcome registered and studied. The occurrence of early neonatal infection was diagnosed by a neonatologist. Diagnosis of the EONI was made using a combination of the clinical signs of infection (hypothermia, respiratory distress, lethargy, hypotonia, irritability, bradycardia) with laboratory findings (total blood count with differential, CRP ≥10 mg/dL) or by positive culture of the urine, cerebral fluid or blood. With the purpose of determining differences in demographic characteristics, the participants were divided in two groups: mothers of newborns without infection (group 1) and mothers of newborns with infection (group 2). With the aim of determining the association of risk factors and neonatal infection, all newborns were divided into two groups based on the presence of the neonatal infection: newborns without infection (group 1) and newborns with infection (group 2).

### Statistical Analysis

aSPSS 17.0 statistical software was used for statistical analysis. Pearson’s chi-square test and the t-test were used as appropriate. Clinical characteristics were compared using descriptive statistics and are presented as percentages, frequencies, and means.

## Results

Two hundred pregnant women complicated with PROM and their newborns were included in the study. The mean age of patients was 27.5 years [standard deviation (SD) ±5.5]. The majority (67%) of the participants were young, aged 20-29 years, primiparous (67.5%), unemployed (82%), had completed secondary level education (44.5%), with middle socioeconomic status (66.5%), and the average number of people per household was revealed as 6.3 (SD±3.7). Although there were no statistically significant differences regarding mean age, family size, and socio-economic status between the two groups, the registered percentage difference regarding parity and employment status was statistically significant (p<0.05) (Table 1). Twenty-six (13%) of the 200 newborns were diagnosed as having EONI. Of the 26 infants with neonatal infection, the highest percentage had pneumonia (46.2%), followed by sepsis (38.5%), systemic inflammatory response syndrome (11.5%), and 1 infant had meningitis. Exitus letalis was registered in two infants (1%). These were newborns of mothers with PROM in less than 37 weeks of gestation and who were found to have neonatal sepsis. The average weight of newborns with regard to registered neonatal infection was 2466.9±853.7 g, whereas in those without neonatal infection it was 3183.2±567.2 g. The difference between the average values was statistically significant (p<0.001). The mean interval from PROM to delivery in patients with registered neonatal infection was 34.7±20.3 hours, and the mean interval in the group without neonatal infection was 23.3±12.6 hours (Table 2). According to t-test the difference was statistically significant (p<0.001).

The data analysis of the high vaginal swab results revealed that out of 61 colonized mothers, 21 had newborns with infection, thus indicating a significant statistical association between maternal genital tract colonization and EONI. The difference between the mean Apgar scores at the 1^st^ and 5^th^ minutes after birth between the groups with and without neonatal infection was statistically significant (p<0.05). There were no significant differences according to the mode of delivery between the groups (Table 2).

## Discussion

EONI remains one of the most serious complications in pregnancies complicated with PROM and poses a major health challenge, especially for developing countries with higher reported rates of early neonatal morbidity and mortality. In this study, out of 200 newborns born following PROM, 13% had EONI. The incidence found in this study is comparable with the results of other studies found in the literature; however, it is important to acknowledge that the literature reveals a wide variation of EONI incidence in pregnancies complicated with PROM.

The findings in this study are in congruence with Asindi et al.^(8)^ who reported an incidence of 14% of neonatal infection after PROM. In contrast, Popowski et al.,^(9)^ in a study among 399 pregnant women complicated with PROM, reported that 4.3% of newborns were diagnosed as having EONI.In a cohort study conducted in China, Wu et al.^(10)^ reported a much greater incidence (25%) of EONI in pregnancies complicated with PROM. According to our study, the observed rate of EONI in pregnancies complicated with PROM indicates a high neonatal infectious morbidity rate compared with the results of more developed countries. Out of all 200 newborns, early-onset neonatal sepsis was proven in 5% of cases. This finding is in agreement with Alam et al.^(11)^ who conducted a cohort study over a five-year period. Authors analyzed neonatal outcome among neonates who had a maternal history of PROM and they reported an incidence of early neonatal sepsis as 4%. In another prospective study among 135 infants born after PROM, the reported incidence of early-onset neonatal sepsis was 8.1%.^(12)^ Similarly, Lee at al.^(13)^ reported an incidence of 6.5% of culture-proven sepsis among neonates born from pregnancies complicated with PROM. This greater incidence of sepsis among infants born after PROM might be due to the fact that these previous studies enrolled newborns born after PROM with a latency period of greater than 24 hours, whereas in our research we included newborns born after PROM for whom the duration of latency period was restricted to a minimum of 1 hour and maximum of 72 hours. In this study, the majority (73%) of infected neonates were born preterm. This higher rate of EONI among preterm infants is expected and is explained by prematurity and associated incomplete maturation of the immune system, which increases the likelihood of infections. The association of birthweight and neonatal outcome is well known. In a recent study, the median birthweight of a group of neonates with EONI was lower (2446 g) than of neonates without infection (3183 g), the difference being statistically significant (p<0.05). This higher rate of EONI among newborns with lower birth weight is expected and is explained by the immaturity of systems of organs including the immune system. This is in accordance with the finding that birth weight is a significant predictor of neonatal outcome and it is inversely related to risk of EONI, as observed in other studies.^(14)^

The interval between PROM and delivery is a factor that may influence maternal and fetal wellbeing; prolongation of the interval from PROM to delivery increases the incidence infection.^(15)^ In recent research, the interval from membrane rupture to delivery ranged from 7 to 70 hours with a mean of 24.8 hours. Our evaluation of PROM to delivery interval in terms of neonatal infection showed a significantly longer IP of 34.7 hours in the group with neonatal infection versus 23.3 hours in the group without neonatal infection. This finding is in accordance with the findings of Herbst and Kallen^(16)^. They reported that the duration of membrane rupture was an independent risk factor for neonatal sepsis and this risk of neonatal sepsis increased independently and nearly linearly with duration of membrane rupture up to 36 hours, with an odds ratio of 1.29 for each 6-hour increase in membrane rupture duration.

A number of studies have highlighted the association between neonatal infection in the first days of life and maternal genital tract colonization. Among the 200 participants included in the present article, high vaginal swab results were positive in 61 (30.5%) cases. Data analysis revealed that out of 61 colonized mothers, 21 had newborns with infection, thus indicating a significant statistical association between maternal genital tract colonization and EONI. Evaluation of the 1^st^ and 5^th^ minute Apgar scores among neonates with and without neonatal infection showed that Apgar scores were statistically significantly lower in the group with neonatal infection compared with group without neonatal infection (p<0.05). Analysis in a recent study identified 1^st^ minute Apgar scores of 5.7±1.5 as a strong risk factor for the development of early neonatal infection, thus this finding supports observation and screening of neonates for possible EONI. In accordance with our results, Hayun et al.^(17)^ reported that Apgar score, gestational age, and weight at birth are risk factors for EONI. In summary, the data reported in the present study are in alignment with the literature from developing countries, demonstrating high neonatal infectious morbidity rates in pregnancies complicated with PROM.

### Study Limitations

The limitation of the present study is its sample size. Future research using larger sample sizes are warranted to replicate these research findings.

## Conclusion

The finding of an EONI rate of 13%, of which 5% were confirmed as early-onset neonatal sepsis, provides additional evidence indicating a high level of EONI among newborns of mothers complicated with PROM.

Risk factors for the development of EONI in pregnancies complicated with PROM are: PROM-delivery interval, low gestational weight and low gestational age at birth, maternal colonization, and low Apgar score. The findings of this study contribute to the larger literature by confirming that neonatal infection in pregnancies complicated with PROM is present as a challenge in Kosovo. Future steps to be undertaken include revision and improvement of antenatal care practices and prophylactic use of antibiotics in PROM.

## Figures and Tables

**Table 1:**
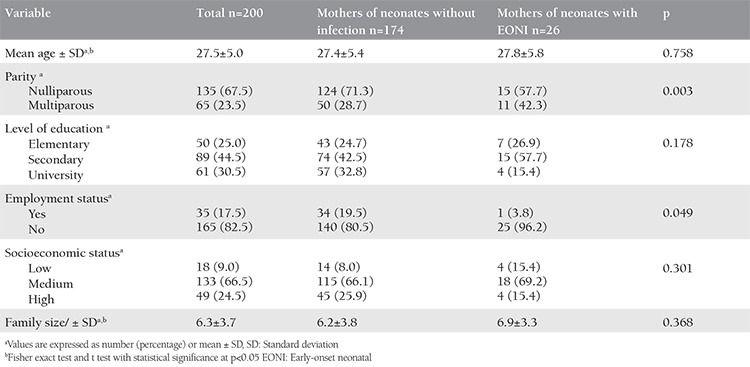
Comparison of maternal characteristics

**Table 2:**
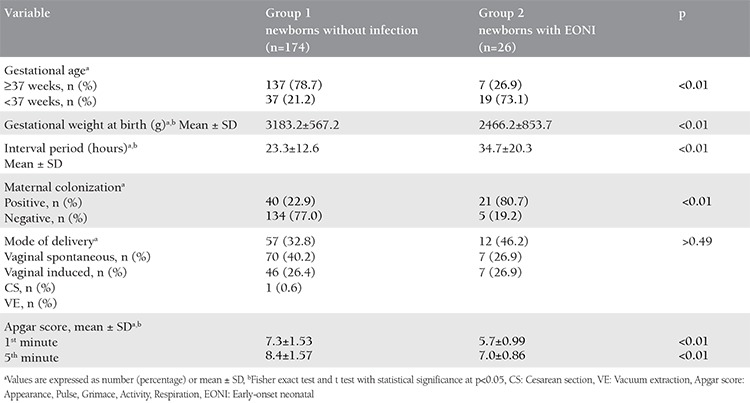
Risk factors and neonatal infection
